# The Role Geomorphic Origin and Agricultural Legacy on Plant Communities and Surface Soil Organic Matter in Salt Marshes

**DOI:** 10.1007/s13157-026-02117-9

**Published:** 2026-09-16

**Authors:** Chance Hines, Laura Duval, Bryan D. Watts, Molly Mitchell

**Affiliations:** 1 Center for Conservation Biology, William & Mary, Williamsburg, Virginia USA; 2 Virginia Institute for Marine Science, William & Mary, Gloucester Point, Virginia USA

**Keywords:** Saltmarsh, Marsh migration, Agricultural legacy, Soil organic matter, Land-use history, Climate change impacts, Carbon sequestration

## Abstract

Saltmarsh habitats provide numerous ecosystem services, including carbon storage, but are eroding at increasingly rapid rates due to ongoing sea level rise. Yet, where space and topography permit, saltmarshes are transgressing into adjacent uplands and counterbalancing the lost area. In these areas, older portions of marshes have accumulated more soil organic matter (SOM) over time than more recently formed marshes, though processes driving this pattern can be complex. Sediment deposition dynamics and plant community can influence SOM with both playing important but interacting roles. We used structural equation modeling to explore how surface SOM and plant communities vary across saltmarshes in the Chesapeake Bay region of Virginia, USA. We evaluated the influence of geomorphic origin (young vs. old), agricultural legacy, and two principal components, PC1, driven by hydrological conditions, and PC2, driven by salinity gradients on surface SOM and plant responses. We found that species-specific plant cover and tree counts were each affected by different combinations of these factors. Surface SOM was greatest in marshes formed prior to 1937, with an agricultural legacy, higher PC1 scores, lower PC2 scores, greater nearby *Phragmites australis* cover, and more nearby snags. We also found important indirect effects, whereby landscape history and environmental predictors influenced surface SOM accumulation through their effects on plants. We hypothesize that elevated surface SOM in areas with an agricultural legacy may result from restricted tidal flooding and reduced inorganic sediment input, conditions potentially created by the presence of berms. However, further investigation is necessary to conclusively address this issue.

## Introduction

Saltmarsh habitat loss is accelerating due to increasingly rapid rates of ongoing sea level rise (SLR) even as new saltmarsh is also rapidly transgressing into uplands where topography is suitable for marsh migration (Ezer and Corlett 2012, Ablain et al. 2019; Fagherazzi et al. 2020). Globally, more saltmarsh has been lost than formed over recent decades, but the rate of marsh creation has compensated for marsh lost to SLR in some regions (Kirwan and Megonigal 2013 , Raabe and Stumpf 2016; Schnieder et al. 2018, Campbell et al. 2022). Marsh loss typically occurs in the oldest portions of marsh via edge erosion (Wasson et al. 2019; Mariotti 2020) while marsh transgression into uplands is a key component of saltmarsh formation, albeit often impeded by barriers such as flood prevention infrastructure or steep terrain (Roman 2017; Miller et al. 2021). However, the implications of recently transgressive marsh replacing long-established marsh are not fully understood. Here, we focus on surface soil organic matter (SOM) as an indicator of recent organic material accumulation, rather than a direct measure of carbon stocks or long-term sequestration.

Maintaining saltmarsh habitat extent is important because it is among the world’s most efficient ecosystems for long-term carbon burial and storage (Pennings and Bertness 2001; Mcleod et al. 2011). Saltmarsh soils include a relatively high proportion of peat, which is a carbon-rich material that consists of partially decomposed organic matter that accumulates in waterlogged and acidic conditions (Drexler 2011). This peat is primarily derived from plant material that grew within the marsh and the ability of marsh soils to accrete peat can vary based on a range of factors including plant species composition, tidal dynamics, sediment deposition, and other environmental conditions (Nyman et al. 2006; Turner et al. 2000). These factors not only influence organic matter accumulation but also determine the marsh’s capacity to adapt to rising sea levels, ensuring its long-term sustainability and its role as a vital carbon sink (Chmura 2013; Stevenson et al. 1986).

Saltmarsh plants are one of the primary drivers of peat formation and both root growth and above-ground biomass contribute (Nyman et al. 2006). Root systems help stabilize the soil and promote vertical accretion by binding the soil and facilitating the accumulation of organic matter while above-ground biomass, including stems and leaves, can contribute to above-ground carbon storage, which can eventually be transferred to the soil as plant material decomposes (Cahoon et al. 2021; Sarika and Zikos 2021). Different plant species may influence the rate of peat accumulation in varying ways due to differences in root depth, growth patterns, and biomass production. Species that have deeper or more extensive root systems may facilitate more substantial soil stabilization and carbon sequestration (Bass et al. 2022). Additionally, marsh plant species composition can affect the overall SOM within the marsh, as species with higher root biomass and slower decomposition rates tend to increase peat accumulation more effectively than those with faster turnover rates.

Saltmarsh soils also include inorganic sediment, like sand, which is deposited from the water column during high tide flooding as well as from adjacent upslope areas following rain events (Pannozzi et al. 2023). Deposition of sediment is important to marsh persistence because it contributes to marsh accretion and areas with greater sediment deposition are better able to keep pace with SLR (Stevenson et al. 1986; Weston et al. 2023). Plant species play a key role in trapping and stabilizing these sediments, particularly through the above-ground biomass, which slows water flow and promotes sediment deposition (Stumpf 1983). The structural complexity of plant communities can enhance the accumulation of inorganic sediments, thus further increasing marsh elevation and resilience against rising sea levels (Kirwan and Megonigal 2013; Granse et al. 2024).

Soils in older portions of marshes typically hold the highest carbon stocks because the proportion of organic matter and soil depths are greater (Mcleod et al. 2011; Theuerkauf et al. 2015). However, these portions of the marsh are most often the outermost and more susceptible to erosion than newly formed marsh along the upland boundary. As a result, marshes can transition from acting as carbon sinks to carbon sources when marsh loss exceeds marsh creation. Even in regions where rapid upland transgression (the landward migration of marshes) compensates for marsh loss, saltmarshes may still become a carbon sink (Smith and Kirwan 2021). This shift can occur if the carbon footprint of the newly created marsh is less than that of the carbon-rich marsh that is lost, especially if the replacement marsh is younger, less developed, or lacks the same peat accumulation.

Barriers to marsh migration vary widely across landscapes, with urban development representing a well-documented obstacle to upland transgression (Enwright et al. 2016). Coastal cities and towns often have seawalls, levees, roads, and other infrastructure that physically prevent saltmarshes from expanding inland in response to SLR, effectively creating a “coastal squeeze” (Silva et al. 2020). In contrast, rural areas generally offer greater opportunities for marsh migration due to the presence of undeveloped land and fewer artificial barriers (Kirwan and Gedan 2019). However, a less recognized but potentially significant challenge to marsh expansion in these regions is the legacy of past agricultural use. In some rural coastal areas, past agricultural lands are surrounded by berms, dikes, or ditches that were historically constructed, presumably, to protect crops from saltwater intrusion (Mora and Burdick 2013). These features persist as the landscape transitions from agricultural to saltmarsh, potentially limiting the ability of marshes to naturally migrate into adjacent uplands even when SLR and tidal dynamics would otherwise allow it. Unlike urban barriers, agricultural berms may be undocumented, partially degraded, or only intermittently breached, creating complex and uncertain conditions for marsh migration as well as plant establishment (Mora and Burdick 2013). Understanding the extent to which these legacy structures act as barriers is a key research need as conservationists and land managers seek to maintain saltmarsh resilience in the face of climate change.

The goal of this study is to assess how marshes of differing geomorphic origin, specifically, recently transgressive marshes (young) versus long-established marshes (old), differ in terms of surface SOM, which represents recent organic material accumulation. Additionally, this study aims to evaluate how physical barriers, specifically berms associated with agricultural legacies, may modulate SOM and plant community composition.

## Methods

### Study Area

**Fig. 1:**
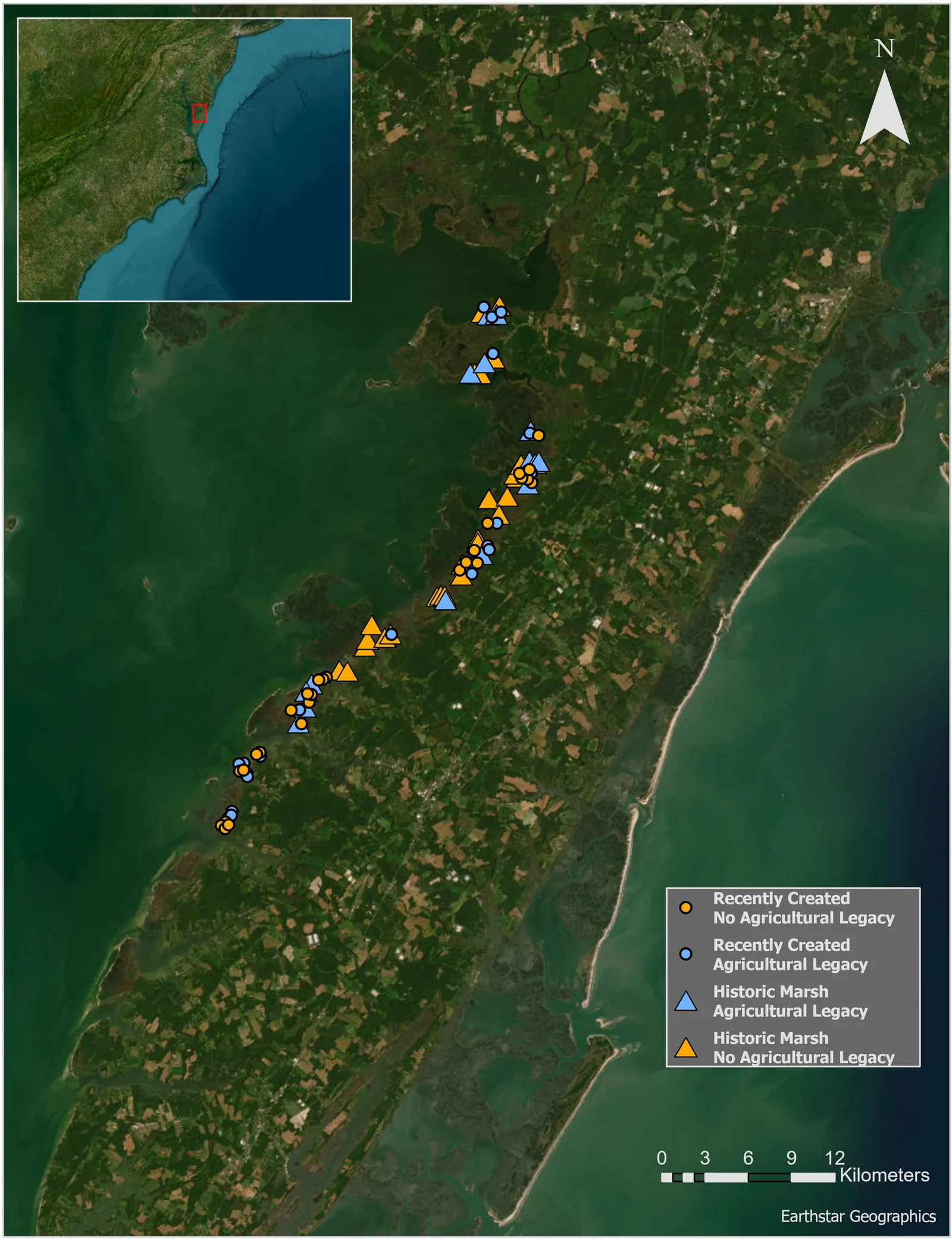
Map of study area and points surveyed on the bayside of the Delmarva Peninsula, Virginia, USA during 2022. Triangles represent marsh patches that were high marsh in 1937 while circles represent points marsh patches that have converted from uplands since 1937. Blue represents points where berms or ditches associated with past agricultural use surround the survey point while orange represents points where no evidence of agriculture exists

The study took place on the Delmarva Peninsula in Virginia, USA (Fig. 1) in saltmarshes along the eastern shore of the Chesapeake Bay (hereafter, eastern shore). The eastern shore is predominantly rural and agriculture has been a prominent industry both historically and in the present day (Gemmill 1926, Lakshmi 2024). This landscape is particularly suitable for studying agricultural legacy effects because many marshes remain bordered by remnant agricultural berms and have experienced relatively little urban development compared to more heavily modified estuaries. Additionally, unlike many marshes farther north, these systems were not extensively mosquito-ditched, allowing historical land-use impacts to be evaluated with fewer confounding alterations. Agricultural activities have historically occurred immediately upslope of saltmarsh but those in lower-lying lands become increasingly unsuitable over time due to saltwater intrusion (Gedan and Fernández-Pascual 2019). Salt water in this area flows from the Atlantic Ocean into the Chesapeake Bay, delivering sediment from offshore sources and influencing marsh development.

### Desktop review

**Fig. 2:**
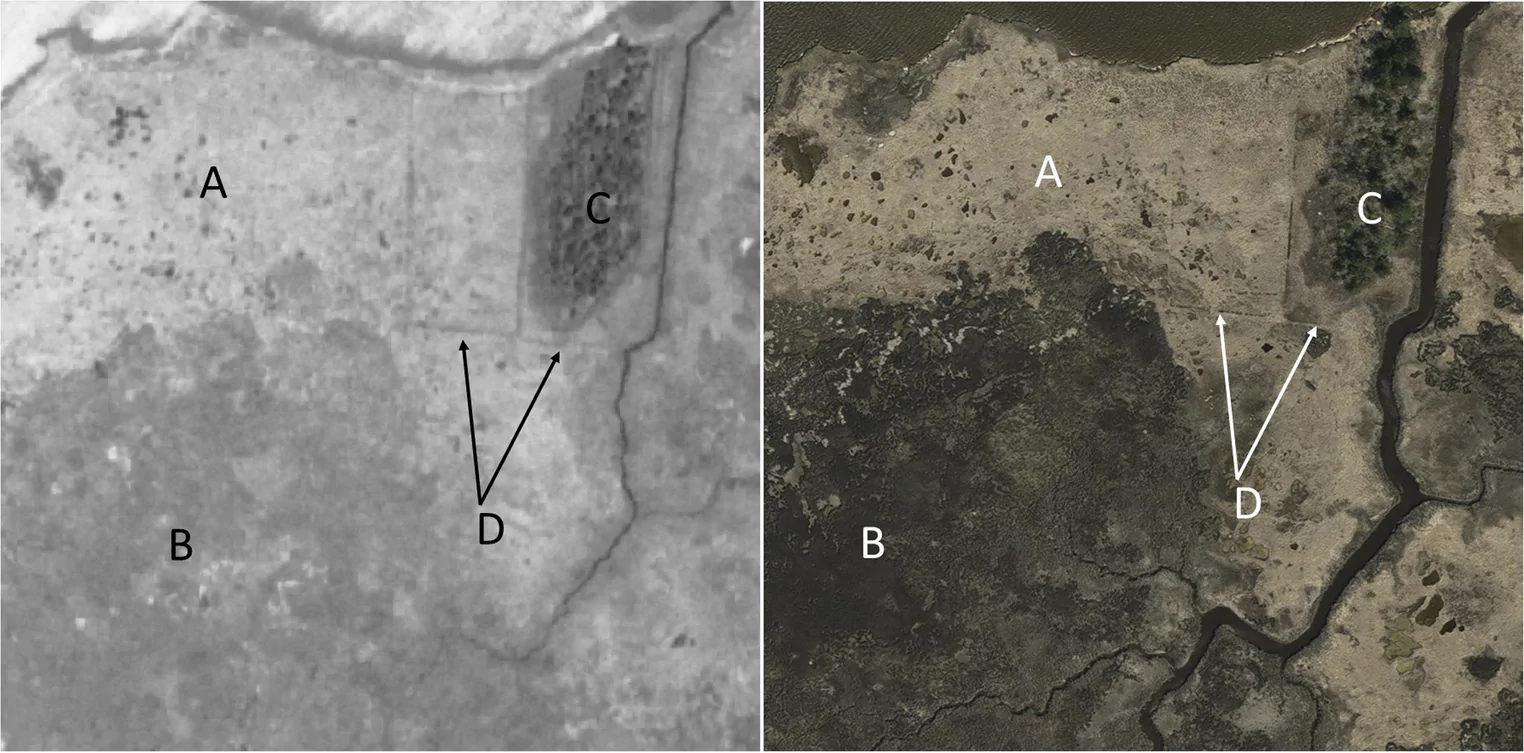
Historic (1937) and current (2021) imagery of a marsh on the bayside of Accomack County, VA. Features on this image include high marsh (A), low marsh (B), forest (C), and two agricultural fields (D) that had already been abandoned by 1937

We used the Virginia Institute for Marine Science (VIMS) shoreline change viewer (Hardaway et al. 2021), which includes aerial photography from 1937 on the bayside to determine whether the historic habitat was high marsh, low marsh, upland, or water and imagery hosted on the VIMS shoreline change viewer from 2021 to classify the current habitat (Fig. 2). The imagery from 1937 was used because it was the earliest consistent imagery, not because it represents an ecological threshold. We considered high marsh habitat to be dominated by short form of Sporobolus alterniflorus (Spartina alterniflora) and Distichlis spicata which were represented by light gray tones in historic imagery and tan tones in modern imagery (A, Fig. 2). We considered low marsh to be dominated by tall S. alterniflorus (Spartina alterniflora) and Juncus roemerianus which was represented by darker tones in historic and modern imagery (B, Fig. 2). Upland habitat was delineated by the presence of fallow fields and actively farmed land or trees and shrubs which are dark in historic imagery and green in modern and can also be identified by their shape in both historic and modern imagery (C, Fig. 2). We also classified survey sites as a former agricultural field if berms or ditches (i.e., agricultural legacy) surrounded a survey point (D, Fig. 2), though some berms may have originally served other purposes, such as protecting residences or low-lying land from flooding, and their precise age and function are often uncertain (Hall et al. 2022). During review of the historical imagery, active agricultural fields consistently exhibited berms or ditches, and no residences or other non-agricultural structures were visible within these fields, supporting our classification approach.

We acknowledge that some uncertainty is inherent in classifying habitats from aerial imagery. For example, short-form *S. alterniflora* can occur at lower elevations than *S. patens* and *D. spicata*, but in aerial imagery, the short-form cannot be reliably distinguished from *D. spicata* or *S. patens*, whereas tall-form *S. alterniflora* is visually distinct. Similarly, some berms may have originally served other purposes, such as protecting residences or low-lying land from flooding, and their precise age and function are often uncertain (Hall et al. 2022) but during review of historical imagery, active agricultural fields consistently exhibited berms or ditches, and no residences or other non-agricultural structures were visible within these fields. Although these features may not perfectly reflect historic marsh conditions, we expect our focus on broader historic habitat categories to remain robust.

### Site Selection 

Survey areas were selected based on our ability to access them from adjacent uplands, sample as many points within a marsh during a single morning as possible, sample both old and new marsh, and include areas with and without an agricultural legacy. We selected 89 points that were placed ≥ 200 m from the nearest neighboring survey point. Additionally, survey points were placed within marshes where the habitat was similar within 50 m, ensuring points were ≥ 50 m from the nearest different-aged marsh or marsh with a different agricultural history.

### Field Protocol 

We collected finer scale habitat predictors between 10 Jun and 31 July 2022 at all points. We visually estimated % cover for all plant species that comprised ≥ 5% cover within 50 m of each survey point as well as the number of living trees and snags within 50 m of each survey point. We quantified the % organic matter in soils by extracting three 7.62 cm diameter, 15.24 cm deep samples at the center of each survey point. The % Soil Organic Matter (SOM) was determined using a loss-on-ignition protocol (Heiri et al. 2001). All cores were stored in a zipper storage bag for two days, transferred to an aluminum tray, dried at 60 °C until their weights became stable, and then muffled at 450 °C for 7 h. The loss-on-ignition method used here provides a reliable and widely used metric for comparing relative differences in SOM among sites, although it is not intended for estimating absolute carbon stocks. 

### Statistical Analyses

We took the mean for the three replicate soil samples from each point and compared percentages of SOM between marshes with and without evidence of past agricultural use with a paired t-test. We also compared those that were recently created and those that existed since the early-mid 20th century with paired t-tests. To evaluate whether newly created marshes occurred at systematically higher elevations than older marshes, we compared mean plot elevation between age categories using Welch’s two-sample t-test, which does not assume equal variances.

We used a structural equation model (SEM) in a Bayesian framework with the ‘brms’ package (Bürkner 2017) in Program R (R Core Team 2023) so that we could estimate both direct and indirect effects of predictors on response variables. Our SEM was intended as an exploratory framework to identify dominant pathways linking environmental gradients, vegetation, and SOM, rather than to establish definitive causal relationships. Within our SEM, we specified 10 individual regressions corresponding to the following response variables: SOM, and plant cover for 8 species, tree counts, and snag counts. We included all plant species individually rather than grouping by functional type because the marsh community is relatively simple, and each species may contribute differently to carbon accumulation. We were interested in plant and SOM responses to predictors that might affect plant occurrence as well as soil composition. Included among these were several likely to covary such as salinity, tidal range, the number of days that tidal flooding exceeded 0.5 m during the study year, surface elevation (± 10 cm, VGIN 2018), and latitude. We calculated tidal range and the number of days during the study year when tidal flooding exceeded 0.5 m using tidal data derived from the nearest NOAA tide gauges and adjusting data using tidal offsets from the CO-OPS Discrete Tidal Zoning Map (NOAA 2017). We tested for covariance using Pearson correlation coefficients and used Principal Component Analysis (PCA) to reduce the dimensionality of covarying predictors after centering and scaling them. Each species-specific plant and tree/snag count regression included the following predictors: The first two principal components, marsh age (pre- or post-1937), and presence of an agricultural legacy at the survey site. The SOM regression model incorporated the same predictors as the plant models but also included plant cover for each species and tree/snag counts to assess their influence on soil carbon accumulation. This allowed us to estimate the indirect effects of the predictors from the plant models on SOM. We also included a random effect for survey route (approximating marsh patch) to account for spatial autocorrelation among nearby survey points. To appropriately model our response variables, we used a beta distribution for SOM, zero-inflated beta distribution for species-specific plant covers, and a Poisson distribution for snag and tree counts. Given the large number of predictors in models, we incorporated a horseshoe prior (df = 1) to shrink the coefficients of uninformative predictors, thereby improving model parsimony and preventing overfitting (Carvalho et al. 2009). Predictor importance was assessed based on the magnitude and direction of posterior median effect sizes and whether 95% credible intervals excluded zero, indicating strong support for an association. Indirect effects were simplified by multiplying the coefficient of each upstream predictor by its proportional contribution in plant or tree/snags models, and combined (direct + indirect) credible intervals were estimated from posterior distributions. Only pathways with 95% CI excluding zero were emphasized in figures and discussion to aid ecological interpretation. For our models, we ran four chains of 4,000 iterations with 2,000 warm-up iterations. Chain convergence was verified by checking R-hat values (all of which were 1.00) and goodness of fit was inspected using posterior-predictive checks.

Indirect effects were calculated by multiplying the coefficients of important predictors in the SOM regression by their relative contributions in upstream models (plant cover and tree count responses). The relative contribution of each predictor was determined by dividing its coefficient by the sum of the absolute values of all predictors in the corresponding upstream model. We used posterior distributions for each model to estimate combined (direct and indirect) credible intervals.

## Results

We surveyed 89 high marsh points including 41 points that were high marsh in 1937 and 48 points converted to saltmarsh since 1937. Mean elevation was not different between groups (T = -0.006_75.96_, *p* = 0.995) and was 0.56 ± 0.07 m ASL at longstanding marshes compared to 0.56 ± 0.13 at more recently formed marshes. Evidence of past agriculture was present in 37 points including 16 (39.0%) that existed prior to 1937 and 21 (43.8%) that formed since then. Most of the agricultural use predated the historic imagery with 87.5% of recently formed marsh being forest in 1937 while 12.5% remained in agricultural use. All of the patches that were in agricultural use in 1937 converted to forest before converting to saltmarsh.

### Environmental Variables

We detected covariance among latitude, salinity, tidal range, tidal flooding frequency and mean elevation. Among these variables, Latitude had positive correlations with tidal range (0.77) and elevation (0.53) but negative correlations with salinity (-0.91) and number of days where tidal flooding exceeded 0.5 m (-0.74). The first two principal components of our PCA explained the majority of variation in the dataset, with standard deviations of 1.64 (32.2% of variance explained) and 0.97 (19%), respectively. PC3-PC5 explained a combined 31.7% of the variance. We included PC1 and PC2 in all downstream models. PC1 primarily captured a gradient related to geographic position and broad-scale hydrological variation, with latitude having a strong positive loading, while elevation and tidal range both had moderate positive loadings and salinity, and flooding frequency had moderate negative loadings. PC2, in contrast, appeared to represent a salinity-driven gradient, with salinity having a strong negative loading, tidal range and elevation both having moderate negative loadings, and tidal flooding having a small negative loading (Table 1).

**Table 1:** Principal component loadings for the first two components of the principal component analsyis

Predictor	PC1	PC2
Latitude	0.550614	0.114735
Salinity	-0.33798	-0.75149
Tidal flooding	-0.48062	-0.12625
Tidal range	0.432812	-0.47218
Elevation	0.405302	-0.42802

### Plant Cover 

**Fig. 3:**
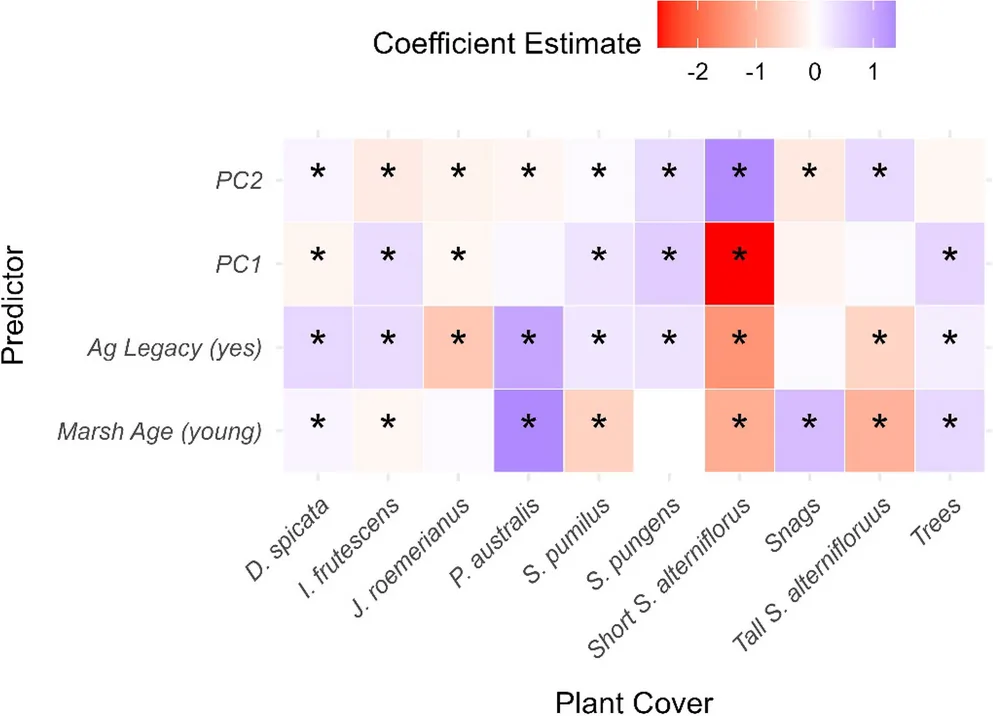
Plant species found on > 10% of survey plots with the number of plots where each was present (n), and the coefficient with 95% credible intervals for predictors in each response-specific regression within our structural equation model. number of survey points (n) that each plant or tree type that was Plant species and tree count responses with the effect size and 95% credible intervals

Eight plant species were observed at >5% of survey locations (Fig 3) and the most common was D. spicata (92.1% of plots, n = 89) while the least common was S. pungens (21.3% of plots). Live trees were observed at 67.4% of points and the average number of trees per plot was 13.3 while snags were observed at 75.3% of plots with a mean of 23.1 per plot.

While our structural equation model (SEM) was used as an exploratory framework rather than to establish definitive causal relationships, it provided insight into dominant pathways linking environmental predictors, vegetation, and SOM. Predictors that were important for species-specific plant covers and tree counts varied by response (Fig. 3). Whether a marsh formed before or after 1937 and whether a site supported an agricultural legacy were important predictors for six plant species, trees, and snags. PC1 was an important predictor for six plant species and living trees while PC2 was an important predictor for eight plant species and snags. Marshes formed since 1937 supported less short and tall *S. alterniflorus*, *S. pumilus*, and *I. frutescens* but supported more *D. spicata*, *P. australis*, trees and snags. Areas with an agricultural legacy supported more *S. pumilus*, *D. spicata*, *S. pungens*, *I. frutescens*,* P. australis*, and trees while they supported less short and tall *S. alterniflorus* and *J. roemerianus*. PC1 positively influenced cover for *S. pumilus*,* S. pungens*, *I. frutescens*, and tree counts while negatively influencing *J. roemerianus*, short *S. alterniflorus*, *D. spicata*. PC2 was positively associated with short and tall *S. alterniflorus*, *S. pumilus*, *D. spicata*, *S. pungens* while negatively influencing *J. roemerianus*, *I. frutescens*, *P. australis*, and snags.

### Soil organic matter

For direct effects within our SEM, SOM was negatively associated with recently formed marshes (-0.78, -1.16:-0.40) and PC2 (-0.17; -0.35:-0.00) and positively associated with PC1 (0.41, 0.20:0.63), snags (0.22, 0.01:0.42), and *P. australis* cover (0.19, 0.02:0.37). Though 95% CI overlapped zero, the absolute coefficient size for agricultural legacy (0.23, -0.09:0.56) was greater than that of all other predictor coefficients except for PC1 and marsh age.

For direct effects within our SEM, SOM was consistently lower in recently formed marshes (-0.78; 95% CI: -1.16 to -0.40) and areas with higher PC2 scores (-0.17; -0.35 to -0.00), and higher in marshes with elevated PC1 scores (0.41; 0.20 to 0.63), greater snag counts (0.22; 0.01 to 0.42), and higher *P. australis* cover (0.19; 0.02 to 0.37). Although the 95% credible interval for agricultural legacy overlapped zero (-0.09 to 0.56), the magnitude of its effect (0.23) was larger than all other predictors except marsh age and PC1, indicating relative importance.

**Fig. 4:**
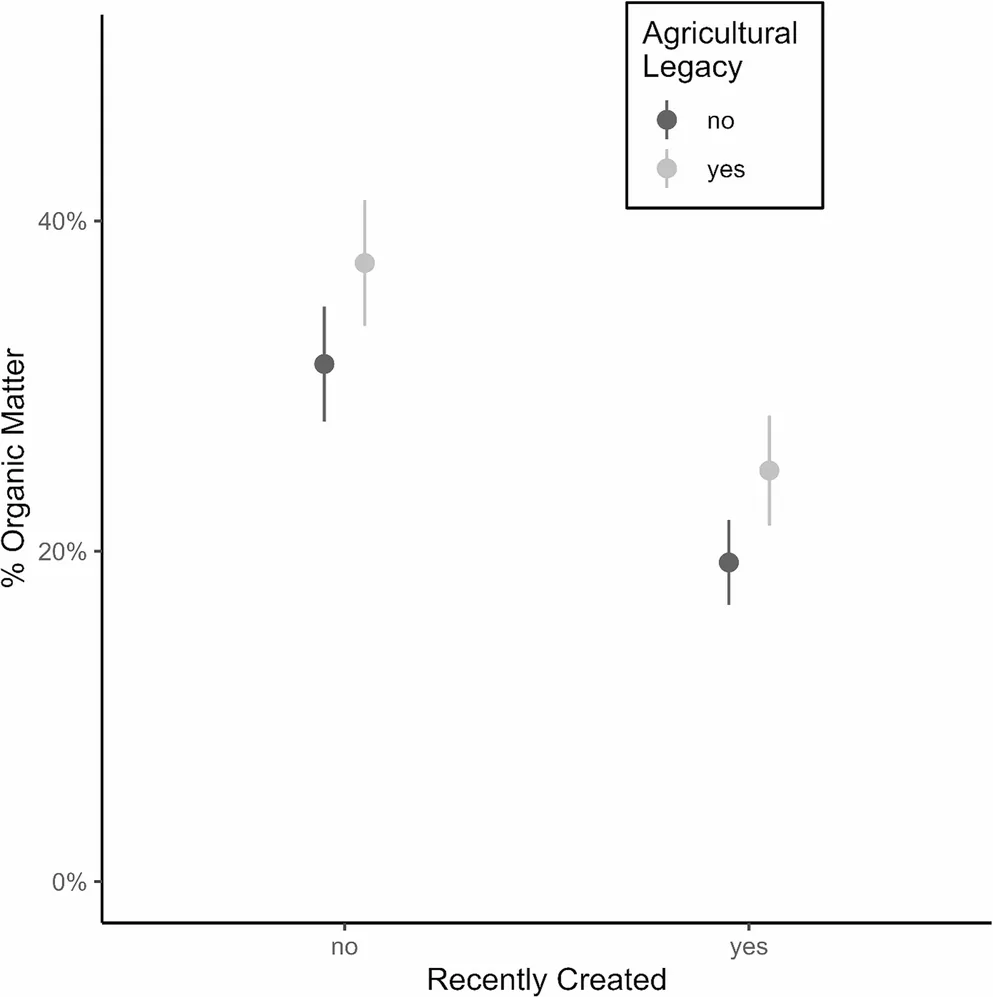
Median posterior predictions for soil organic matter at marshes that formed before and after 1937 in areas with and without an agricultural legacy. Error bars represent standard deviation of predicted mean values across posterior draws

SOM was also shaped by indirect effects mediated through snag counts and *P. australis* cover. For recently created marshes, positive indirect effects partly offset negative direct effects, reducing the magnitude by 29.5% (-0.54; -0.89 to -0.17). For areas with an agricultural legacy, positive indirect effects increased the overall coefficient by 36.6% (0.32; -0.01 to 0.65). Indirect effects for PC1 included a small positive contribution from snags and a slight offset from *P. australis*, resulting in an overall 10–36% increase in effect size (0.46; 0.24 to 0.66). Overall, predicted SOM was highest in older marshes with an agricultural legacy (Fig. 4).

## Discussion

We expected older marshes to exhibit greater surface organic matter (SOM) due to longer periods of sediment accumulation and organic matter inputs. Our results largely aligned with those expectations and we found that marsh surface soils in earlier stages of development, specifically those formed since 1937, tend to have lower SOM. Marsh age was the strongest predictor of SOM in our analysis, consistent with research indicating that marshes accumulate organic matter over time and older marsh patches have more SOM (Mcleod et al. 2011; Theuerkauf et al. 2015). This pattern supports the idea that newly formed marshes do not provide the same carbon storage potential as older marshes.

Marsh age also played an important role in shaping the plant communities at our study sites. Four plants were more abundant at older marshes, though two of these were distinct forms of the same species (short and tall forms of *S. alterniflorus*), while two were more abundant in newer marshes. The greater abundance of both *S. alterniflora* forms in older areas likely stems from their high tolerance of both inundation and saline growing conditions (Bertness et al. 1992 A). These older marsh sections are typically located closer to the marsh–water interface, where longer inundation periods lead to more saline soils than those found near the upland edge and *S. alterniflora* can outcompete species less tolerant of the saline hydrological conditions.

Competition likely plays a role in shaping other species distribution as well. We believe the association between *D. spicata* and marsh age was driven by interspecific interactions. *D. spicata* has similar salt and inundation tolerances as *S. pumilus* but was more abundant in more recently formed marsh while *S. pumilus* was more abundant in older marsh, possibly because *D. spicata* often colonizes disturbed areas but is outcompeted by *S. pumilus* over time (Crain et al. 2008). *I. frutescens* was more abundant in older portions of the marsh possibly because those portions of the marsh are more saline, suppressing other shrubby species. Saline growing conditions restrict other shrubs like *M. cerifera* and *B. halimifolia* that outcompete *I. frutescens* in the more recently formed, less saline portions of the marsh (Bertness et al. 1992B).

The only other plant that was more abundant in more recently formed marsh was *P. australis* and the effect of marsh age was approximately 10 times as great as for *D. spicata*. *P. australis* is an exotic invasive in Virginia and is known to competitively exclude other plant species by forming thick monotypic stands (Hazelton et al. 2014). While *P. australis* is tolerant of prolonged inundation, it is not as tolerant of saline conditions as other plants in our study (Hellings and Gallagher 1992; Lonard et al. 2013). As marshes develop and mature inland, their elevation relative to tidal flooding can decrease, leading to more frequent and prolonged periods of inundation as well as more saline conditions, which limits *P. australis*’ ability to persist, potentially reducing its dominance over time (Vasquez et al. 2006). This pattern was also evident for trees, which often die as a result of increased inundation and salinity (Pezeshki et al. 1990), that were more abundant in more recently formed marshes. Our contention that salinity is driving these processes is supported by our finding that the principal component most strongly driven by salinity (PC2) negatively influenced snag counts and *P. australis* while positively influencing tall *S. alterniflorus*, the most salt-tolerant of plants at our site (Bertness et al. 1992 A).

Our PCA results highlighted more than just the effect of salinity on plant communities as both PC1 and PC2 were important in predicting SOM. The first component (PC1), which was primarily driven by a geographic and hydrological gradient, had a positive effect on surface SOM concentration, while the second (PC2), a gradient most strongly driven by salinity, had a smaller negative effect. The strong positive loading of latitude on PC1 suggests that geographic position may be influencing SOM concentration, potentially serving as a proxy for factors not explicitly included in the PCA, such as sediment supply. Lower-latitude sites in our study area likely receive greater inputs of inorganic sediment due to proximity to the mouth of the Chesapeake Bay, where large deposits of sand flow northward up the eastern shore of the Bay (Colman et al. 1988). Increased inorganic sediment supply could dilute SOM, leading to lower SOM concentrations at these sites but could also increase the potential for the marsh to accrete and keep pace with SLR. Meanwhile, the negative effect of PC2 suggests that higher salinity and reduced tidal range may create conditions less favorable for organic matter retention, reinforcing the importance of broader environmental gradients in shaping marsh soil properties.

The habitat in former agricultural fields appear to be particularly influenced by the interacting dynamics captured in our principal components, potentially because legacy berms act as partial barriers to tidal exchange, restricting saltwater intrusion while simultaneously impounding freshwater within the marsh interior. This hydrological modification may dampen the salinity-driven effects observed in PC2, which is consistent with the persistence of *P. australis* and trees in these areas by limiting their exposure to frequent tidal flooding and high salinity. However, by reducing tidal exchange, these same features may also be associated with reduced sediment deposition, particularly the input of inorganic material that typically accompanies tidal flooding (Kirwan and Megonigal 2013). Given that PC1 was positively associated with SOM concentration, areas with greater tidal exchange and greater potential for sediment deposition may experience a dilution effect, where greater inorganic inputs decrease the relative concentration of SOM. While a greater concentration of surface SOM may align with higher carbon storage, this accumulation may be offset by hydrological conditions that do not favor long-term carbon sequestration, such as extended periods of standing water or prolonged anaerobic conditions (Mitsch and Gosselink 2015). These findings align with studies showing that hydrologic alteration can create spatial variability in marsh structure and function, with impounded areas often exhibiting distinct plant communities and soil characteristics compared to tidally connected marshes (Watson et al. 2017).

**Fig. 5:**
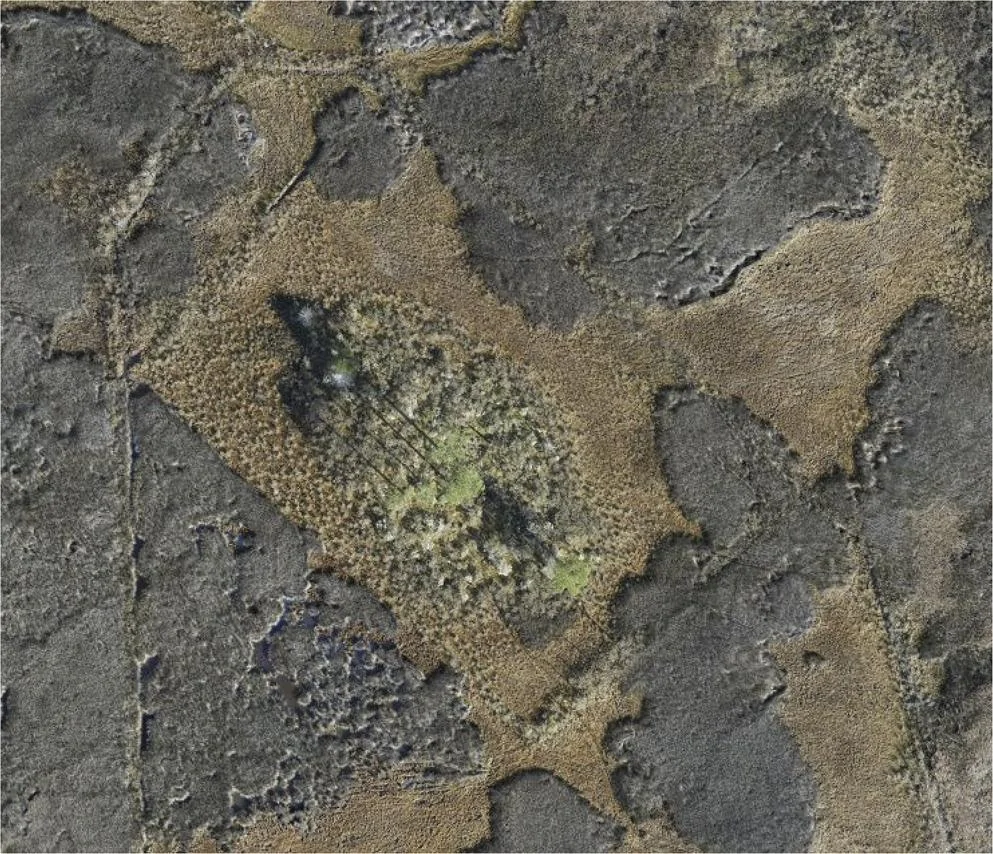
Aerial imagery of an abandoned agricultural field converting to saltmarsh in Accomack County, Virginia, USA. The middle of the field still supports shrubs and a handful of trees. The field is surrounded by berms and ditches that have inhibited colonization within the abandoned field by *J. roemerianus*, most noticeably along the southwest border. Shrubs can also be seen growing along the southernmost portion of the elevated southeast berm between the saltmarsh vegetation

In addition to altering tidal exchange and sediment deposition, agricultural legacy structures may also influence plant community dynamics by creating physical and hydrological barriers to colonization. By restricting saltwater intrusion and maintaining areas of lower salinity, berms could facilitate the persistence of *P. australis*, which thrives in conditions with reduced tidal influence. At the same time, the slightly elevated topography of berms could provide refugia for trees and snags, allowing them to persist in an otherwise flood-prone landscape. These berms, along with the ditches often found adjacent to them, may also act as barriers to marsh plant colonization across the broader landscape. Within the bermed areas, species that spread through rhizomes, like *P. australis*, may persist and expand but the berms limit their ability to spread beyond the berms. This pattern was frequently observed, with distinct plant communities occurring on either side of the berms (Fig. 5). Such hydrologic and physical constraints highlight the lasting influence of past land use on marsh recovery and underscore the importance of considering legacy structures in coastal wetland management.

While the influence of berms and ditches associated with agricultural legacy on tidal exchange and plant community structure provides a compelling explanation for observed patterns, other factors related to past agricultural practices may also contribute to differences in SOM. Agricultural practices such as grazing, tilling, and planting can alter soil structure, organic inputs, and microbial communities in ways that continue to shape marsh soil formation (Wang et al. 2015). However, these practices are generally associated with lower SOM compared to areas that were never cultivated (Burden et al. 2013; Elschot et al. 2013; Wang et al. 2015). Additionally, areas that were never cultivated may have supported forests for longer periods before transitioning to marsh, potentially influencing SOM accumulation through differences in initial soil properties. Again, our findings were not consistent with the expected pattern because, typically, soil carbon is greater in older forests (Paul et al. 2002).

As saltmarsh transgression accelerates due to rising sea levels, understanding how past land use influences carbon storage is crucial for accurately predicting future changes. The ability of saltmarshes to sequester and retain carbon as they migrate into former uplands depends on factors like sediment supply, plant community composition, and soil characteristics, all of which are shaped by historical land use. Whether agricultural legacies promote or hinder carbon sequestration, these past modifications will interact with ongoing environmental changes to influence marsh resilience. Given the rapid pace of these changes, assessing how these factors impact long-term carbon storage is essential for managing coastal wetlands as effective carbon sinks. While management options for saltmarsh transgression into uplands are limited, targeted berm breaching could serve as a useful experimental manipulation to evaluate how hydrologic reconnection affects sediment deposition, vegetation composition, and surface soil development, providing valuable insights into strategies for enhancing carbon sequestration.

Saltmarsh transgression into upland habitats is primarily driven by sea level rise, though the process can be modulated by large pulses of sea water inundation as well as the landscape into which the saltmarsh is transgressing (Fagherazzi et al. 2019). In a saltmarsh transgression scenario driven by steady sea level rise and in absence of pulses of sea water and upslope barriers, SOM accumulation is driven by high carbon burial rates as marsh grasses overtake the upland habitat and existing organic matter degradation slows following inundation (Van Allen et al. 2021). After the uplands convert to saltmarsh, the rate of carbon accumulation slows and is largely based on the ability of marsh plants to persist, anchor the marsh substrate, and trap sediment suspended in the water column (Ford et al. 1999; Goodwin and Mudd 2019). Without accumulation of sediment, vertical accretion is inhibited, leading to eventual marsh degradation and erosion (Baranes et al. 2022).

For some marshes, pulses of sediment, like those associated with infrequent lunar and storm influenced tides, are necessary (Baranes et al. 2022; Goodwin and Mudd 2019) and marsh persistence is compromised when barriers to sediment supply exist (Fagherazzi et al. 2020; Enwright et al. 2016). We believe that berms associated with past agricultural practices act as a barrier and inhibit accumulation of inorganic sediment that is deposited onto the marsh platform from the water column. This is in line with our finding that surface SOM was greater in former agricultural fields than that found in saltmarsh without abandoned farm infrastructure, but this does not necessarily indicate a greater capacity for carbon sequestration but rather a different balance of sediment composition influenced by historical land use practices. The berms associated with past agricultural use may have initially been erected as a measure of flood defense and may continue to alter hydrology (Hall et al. 2022). Many of these berms have fallen into disrepair and include breaches but retain the majority of their structure so that tidal flow and associated sediment deposition may still be restricted. Soils on the landward side of berms within other Chesapeake Bay saltmarshes are typically lower with shallower organic horizons than soils on the bayside of the berm (Hall et al. 2022), supporting this notion. Unfortunately, we did not age the soils and the soil cores we extracted were only 6” deep so we were unable to quantify depth of organic layer. However, the more highly concentrated organic material within the topmost 6” is consistent with a reduced capacity for accreting inorganic sediment. If our observations reflect reduced sediment input, this could limit the overall growth and vertical accretion of these marshes if deeper soil layers than we measured are also affected, potentially impacting their long-term carbon sequestration potential. Without new sediment deposits, the marshes may struggle to keep pace with sea level rise, ultimately affecting their ability to function as effective carbon sinks. If these berms inhibit sediment deposition, the influence of berm height and the number of breaches in the berm may modify its influence on sediment deposition and warrants further investigation.

Our finding that recently converted marshes had lower surface-level SOM than those that were already marsh prior to the early-mid 20th century could have significant implications for carbon sequestration. Saltmarshes are among the world’s most efficient ecosystems for sequestering carbon but can serve as a sink when historical carbon stocks (i.e., SOM) are eroded faster than carbon can be accumulated in a marsh (Mcleod et al. 2011; Theuerkauf et al. 2015). Carbon stocks are relatively low in most upland soils adjacent to marshes, rapidly increase following transgression, and continue increasing at slower rates as they age so the oldest portions of marshes often hold the most carbon stock (Miller et al. 2022; Langston et al. 2022). The relatively rapid rate of saltmarsh loss and creation may be contributing to an overall net loss of carbon stocks that Chesapeake Bay saltmarsh provides as older marsh is increasingly being replaced with more recently transgressed marsh.

Differences in vegetation communities found at our study sites support the distinction between newly created and longstanding marshes and that an agricultural legacy alters the dynamics of a marsh long after agriculture is abandoned. Unsurprisingly, both living and dead trees were more likely to be found in recently created marshes. Trees, particularly *P. taeda*, can persist within and adjacent to saltmarsh for a number of years after the frequency and severity of tidal inundation becomes too severe for the recruitment of young trees (Kearney et al. 2019) while snags can persist for decades after tree death (Kirwan and Gedan 2019). Several plant species were also affected by the recency of marsh creation and presence of an agricultural legacy though the underlying mechanisms behind these trends may vary. For instance, *Phragmites australis*, which is less tolerant of saline conditions than other common saltmarsh plants (Vasquez et al. 2006), likely benefits from suppressed tidal inundation, which creates fresher, more suitable growing conditions. For some plant species, the berms, which we propose act as barriers to tidal inundation, may also impede rhizomatic plant colonization. For example, *J. roemerianus* and both forms of *S. alterniflorus* primarily spread through rhizomatic growth and all had a negative relationship with an agricultural legacy. Notably, *J. roemerianus*, which has expanded significantly throughout the region in recent decades, shows stark differences in establishment on either side of abandoned berms in some patches (Fig. 5). These barriers may favor the persistence of other marsh grasses, such as *Distichlis spicata*, as indicated by its positive relationship with agricultural legacies. A third way in which the agricultural legacy may continue to affect plant cover is that the berms may provide refugia to shrub species (Fig. 5). This notion is supported by the positive relationship between an agricultural legacy and *I. frutescens*, which is less likely to be found in the lower, more saline portions of the marsh (Bertness et al. 1992B). While these patterns are consistent with the hypothesized influence of berms on plant communities, we emphasize that they represent plausible mechanisms rather than proven causal effects.

## Conclusion

As SLR rates continue increasing we expect saltmarsh attributes to continue shifting toward those found in more recently created marshes, specifically changes in plant community composition and surface-level SOM. Our results suggest that both marsh age and agricultural legacies may continue to shape vegetation structure, hydrology, and SOM decades after active use has ceased. Hydrologic connectivity may be a key mechanism underlying these patterns. Berms associated with former agricultural use may alter tidal exchange and salinity regimes, potentially influencing vegetation composition and surface SOM dynamics. However, while we observed differences in surface SOM among marsh types, the implications for long-term carbon sequestration remain uncertain because deeper soil layers were not directly measured. Berm breaching could serve as an experimental management technique to evaluate how restoring hydrologic connectivity affects plant communities and sediment deposition. Such experimental approaches would improve understanding of how legacy structures interact with sea-level rise to shape marsh plant communities and carbon sequestration rates. Recognizing the persistent influence of past land-use and tidal exchange is essential for interpreting marsh responses to ongoing environmental change. 

## Data Availability

The datasets generated during and/or analyzed during the current study are available from the corresponding author on reasonable request.
